# Emergence of a novel reassortant H3N6 canine influenza virus

**DOI:** 10.3389/fmicb.2023.1186869

**Published:** 2023-05-11

**Authors:** Bo Meng, Hailing Li, Chong Feng, Weiwei Guo, Yali Feng, Dawei Zhu, Hualan Chen, Ying Zhang

**Affiliations:** ^1^Key Laboratory of Livestock Infectious Diseases, Ministry of Education, Key Laboratory of Zoonosis, College of Animal Science and Veterinary Medicine, Shenyang Agricultural University, Shenyang, China; ^2^Agricultural Development Service Center of Liaoning Province, Shenyang, China; ^3^State Key Laboratory of Veterinary Biotechnology, Harbin Veterinary Research Institute, CAAS, Harbin, China

**Keywords:** canine influenza virus, H3N2, H5N6, reassortant, replicative ability

## Abstract

Although the natural hosts of avian influenza viruses (AIVs) are wild birds, multiple subtypes of AIVs have established epidemics in numerous mammals due to their cross-species spillover. Replication and evolution in intermedia mammalian hosts may facilitate AIV adaptation in humans. Because of their large population and intimacy with humans, dogs could act as such an intermedia host. To monitor the epidemiology of canine influenza viruses (CIVs) in Liaoning, China, we performed three surveillances in November 2018, March 2019, and April 2019. Five H3N2 and seven novel H3N6 CIVs had been isolated. Since the N6 neuraminidase (NA) genes were clustered with the H5N6 AIV, there is a high possibility that these H3N6 CIVs were generated from a H3N2 CIVs and H5N6 AIVs reassortment case. In addition, the H3N6 CIV showed increased mammalian adaptation ability compared to all the H3N2 strains in both *in vitro* and *in vivo* studies. Even though isolated 3 months later, the March 2019 isolated H3N2 viruses replicated more efficiently than the November 2018 isolated viruses. Our study indicated that H3 CIVs were undergoing an evolution process, through both genetic mutations and gene reassortment, at an incredible speed.

## 1. Introduction

Influenza A virus (IAV), a member of the family *Orthomyxoviridae*, contains eight single-stranded negative-sense RNA segments that encode more than 15 viral proteins (Liu et al., 2020). A total of 18 hemagglutinin (HA) subtypes and 11 neuraminidase (NA) subtypes have been identified in IAV, except H17N10 and H18N11, all of which emerge in avian strains. The natural reservoirs of IAVs are migratory waterfowl, but certain IAV lineages may infect diverse species, including domestic poultry, humans, and other mammals (Krammer et al., 2018).

The H3N2 influenza virus is a major cause of human epidemics annually. H3N2 viruses are also commonly detected in other animals, where they undergo adaptive evolution that facilitates cross-species transmission. The H3N2 avian influenza virus (AIV) can infect and transmit among mammals, posing a potential pandemic threat (Guan et al., 2019; Zhang et al., 2021). H3N2 AIV entered the canine population and formed the canine influenza virus (CIV) lineage in around 2006. Since then, H3N2 CIVs have been isolated in Thailand (Bunpapong et al., 2014) and other areas of Southeast Asia (Sun et al., 2017). In 2015, H3N2 CIVs emerged in the United States (Watson et al., 2017). A new antigenic clade of H3N2 CIV emerged in China in 2016 (Lyu et al., 2019) and became dominant since then (Li et al., 2021).

Dogs can act as a “mixing vessel” of the influenza virus, because they express both human-like and avian-like receptors (Wasik et al., 2017). A large number of studies have shown that dogs could be naturally infected by different origins of influenza viruses, such as human H1N1 and H3N2, swine H1N1, avian H5N1, H5N2, H6N1, H9N2, and H10N8 viruses (Lin et al., 2015; Sun et al., 2017). When multiple influenza virus strains co-infect a dog, a novel reassortant could be generated by gene segments exchanging between influenza viruses. Novel CIVs had been derived in this way from the recombination between H3N2 CIV and the 2009 pandemic H1N1 virus (pdm/09) and swine H3N2 and swine H1N1 viruses (Song et al., 2012; Chen et al., 2018). Given the closeness of dogs and humans, the epidemic and evolutionary statuses of CIVs deserve attention.

Liaoning Province has the largest pet dog breeding and trading base in China. Numerous dogs are distributed daily from Liaoning to other provinces of China. In this study, we investigated the epidemic situation of CIVs and analyzed the biological properties of different isolates in Liaoning.

## 2. Materials and methods

### 2.1. Ethics statement

Mice experiments were approved for use by the Animal Experimentation and Laboratory Animal Welfare Committee of Shenyang Agricultural University (No. 202106009, approved on 8 March 2021).

### 2.2. Sample collection and virus isolation

Active CIV surveillance studies were conducted from November 2018 to April 2019. Dog nasal swab samples were collected from a large-scale animal shelter in Liaoning Province, China. The swab samples were separately put into 1 mL of phosphate-buffered saline (PBS) containing penicillin (2,000 U/mL) and streptomycin (2,000 μg/mL). Viral RNA was extracted from each sample using the TIAN amp Virus DNA/RNA Kit (TIANGEN Biotech, Beijing, China). Reverse transcription-PCR (RT-PCR) was performed using primers targeting the influenza virus matrix (M) gene (the primer sequences are available upon request). The PCR-positive samples were inoculated into 10-day-old specific pathogen-free embryonated chicken eggs (Harbin Weike Biotechnology Development Company, Harbin, China). The inoculated eggs were incubated at 37°C for 72 h. Then, the allantoic fluid was collected for the hemagglutination test, and the positive ones were reserved at −80°C as viral stock.

### 2.3. Whole-genome sequencing and phylogenetic analysis

The isolated viruses were applied to whole-genome sequencing and phylogenetic analysis. The viral RNA was extracted using the TIAN amp Virus DNA/RNA Kit (TIANGEN Biotech, Beijing, China). The RT-PCR test was carried out with primers for each viral gene (the primer sequences are available upon request). Viral genomes were sequenced by Comate Bioscience Company Limited (Changchun, Jilin, China). For phylogenetic analysis, reference sequences of canine, avian, swine, equine, mink, and human influenza viruses were downloaded from three main influenza virus databases: the NCBI influenza virus database, the GISAID influenza database, and the Influenza Research database (Supplementary Table S1). The unrooted tree was constructed using the maximum-likelihood (ML) method with MEGA X (https://www.megasoftware.net) with 1,000 bootstrapreplicates.

### 2.4. Viral replication kinetics *in vitro*

To evaluate the replicative ability of the CIVs *in vitro*, Madin–Darby canine kidney (MDCK) cells were infected with 10^2^ 50% tissue culture infectious doses (TCID_50_) of each virus with volumes of 100 μl. The infected cells were incubated at 37°C for 72 h with Dulbecco's modified Eagle's medium containing 1 μg/mL of tolylsulfonyl phenylalanyl chloromethyl ketone (TPCK)-treated trypsin. The supernatant was collected every 12 h and titrated in MDCK cells.

### 2.5. Replication ability in mice

To evaluate the replicative ability of the CIVs *in vivo*, eight 6-week-old and three 4-week-old female BALB/c mice (Changsheng Biotechnology, Liaoning, China) were inoculated intranasally with each CIV as described previously (Shi et al., 2017). Each mouse was infected intranasally with 10^6^ 50% egg infectious doses (EID_50_) of each virus with volumes of 50 μL. Three mice from each group were euthanized on day 3 post-infection (p.i.), and their nasal turbinates, lungs, brains, spleens, and kidneys were collected for viral titration. The remaining five mice were observed for clinical symptoms. Their body weights were measured every day until day 14 p.i. Five 6-week-old mice were infected intranasally with a 50 μl volume of PBS as the mock groups.

### 2.6. Receptor-binding property analysis using hemagglutination assays

Receptor-binding property is a key determinant of the mammalian adaption of the influenza virus (Gao et al., 2009; Zhang et al., 2012). Hemagglutination assays using resialylated chicken red blood cells (cRBCs) and sheep red blood cells (sRBCs) were performed to analyze the receptor-binding property of the CIVs. The surface of cRBCs expresses both human-like α2,6 and avian-like α2,3 sialic acid receptors (Ito et al., 1997), while sRBCs only contain avian-like α2,3 sialic acid receptors (Medeiros et al., 2001). The α2,3 sialic acids were removed from the cRBCs' surface by incubating with α2,3-sialidase (Takara, Dalian, Liaoning, China). The desialylation cRBCs were generated by incubating with Vibrio cholerae neuraminidase (VCNA, Roche, San Francisco, CA, United States). The desialylation cRBCs were used as the negative control. The A/Sichuan/1/2009(H1N1) and A/chicken/Hebei/3/2013(H5N2), which bind to α2,6-cRBCs and α2,3-cRBCs, respectively (Shi et al., 2017), were used as controls.

### 2.7. Statistical analysis

Statistical analysis between different groups was performed using a one-way analysis of variance (ANOVA) test via the GraphPad Prism version 8.0 (Graph Pad Software Inc., CA, USA). A difference with a value of *p* < 0.05 was considered statistically significant, while *p* < 0.01 was considered highly statistically significant.

## 3. Results

### 3.1. Virus isolation

Three active CIV surveillance studies were conducted on 26 November 2018, 2 March 2019, and 30 April 2019. A total of 534 nasal swab samples were collected from dogs kept in a large-scale animal shelter in Liaoning Province that houses multiple kinds of animals including dogs, cats, swine, and birds. The swab samples were tested for the influenza virus M gene by RT-PCR. In total, 60 samples were positive. The CIV-positive rate in this animal shelter was 11.24% (Table 1). The positive samples were inoculated into embryonated specific-pathogen-free eggs for CIV isolation, which resulted in 12 CIVs being isolated.

**Table 1:** Surveillance of CIVs^y^ in Liaoning Province, China, from 2018 to 2019.

**Collection date (mm/dd/yy)**	**Number of samples**	**CIV-positive rate % (positive/total)**
11/26/18	163	11.04 (18/163)
03/02/19	134	10.45 (14/134)
04/30/19	237	11.81 (28/237)
Total	534	11.24 (60/534)

^†^CIVs, canine influenza viruses.

### 3.2. Phylogenetic analysis

**Figure 1:**
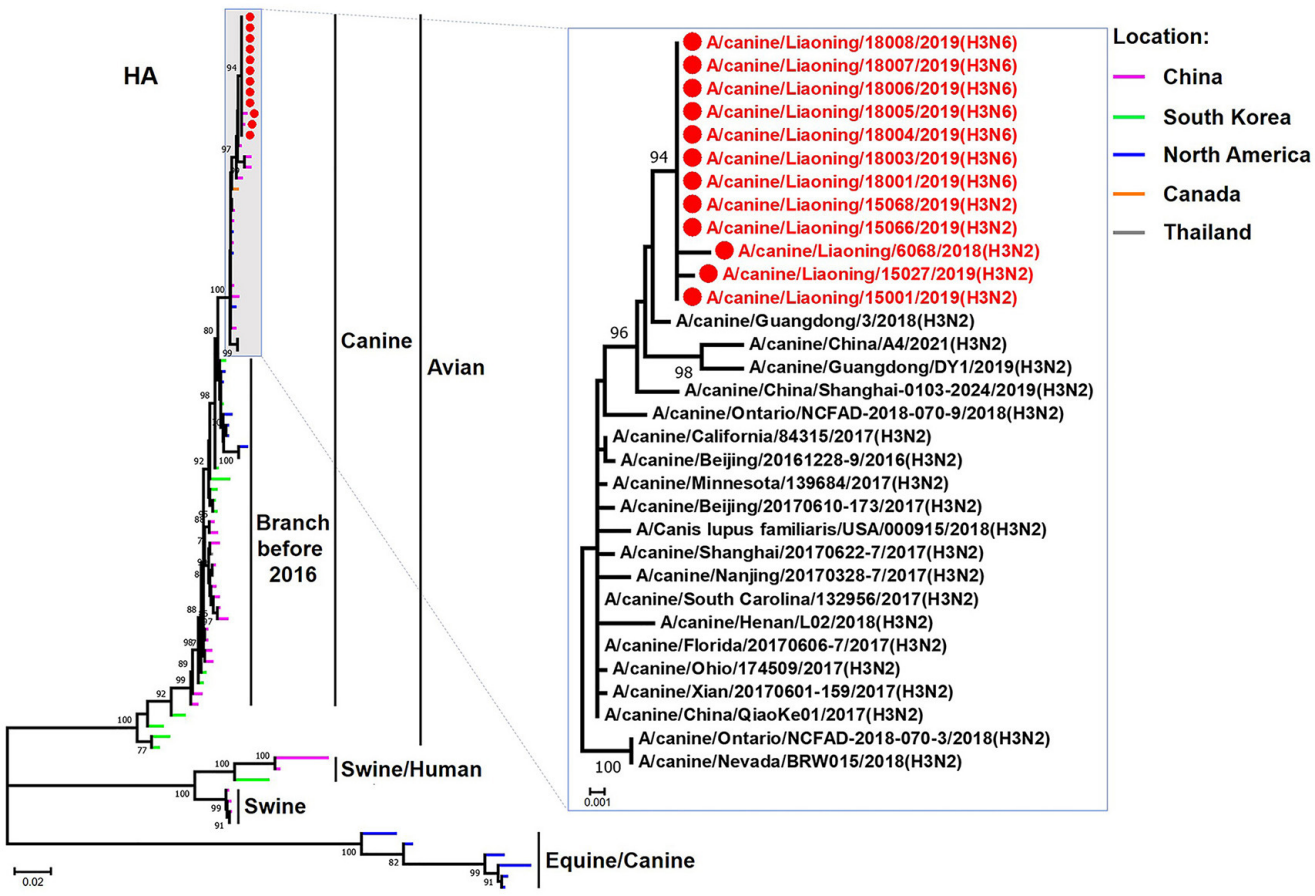
Phylogenetic analysis of the HA genes of H3N2 and H3N6 CIVs. It is based on nucleotide positions 30–1,730 for HA. Isolates in this study are colored red. The scale bar indicates the number of nucleotide substitutions per site.

**Figure 2:**
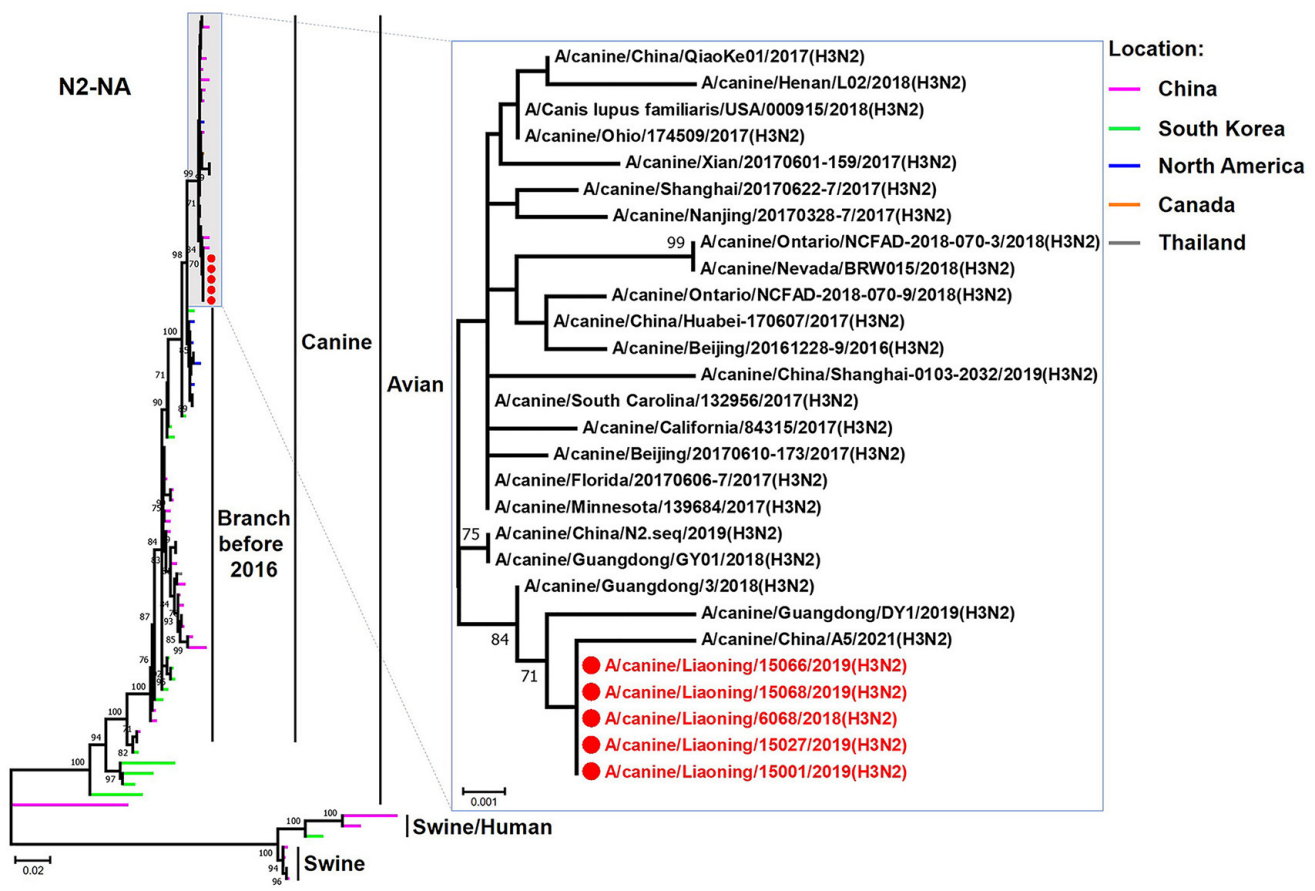
Phylogenetic analysis of the NA genes of H3N2 CIVs. It is based on nucleotide positions 20–1,429 for NA. Isolates in this study are colored red. The scale bar indicates the number of nucleotide substitutions per site.

**Figure 3:**
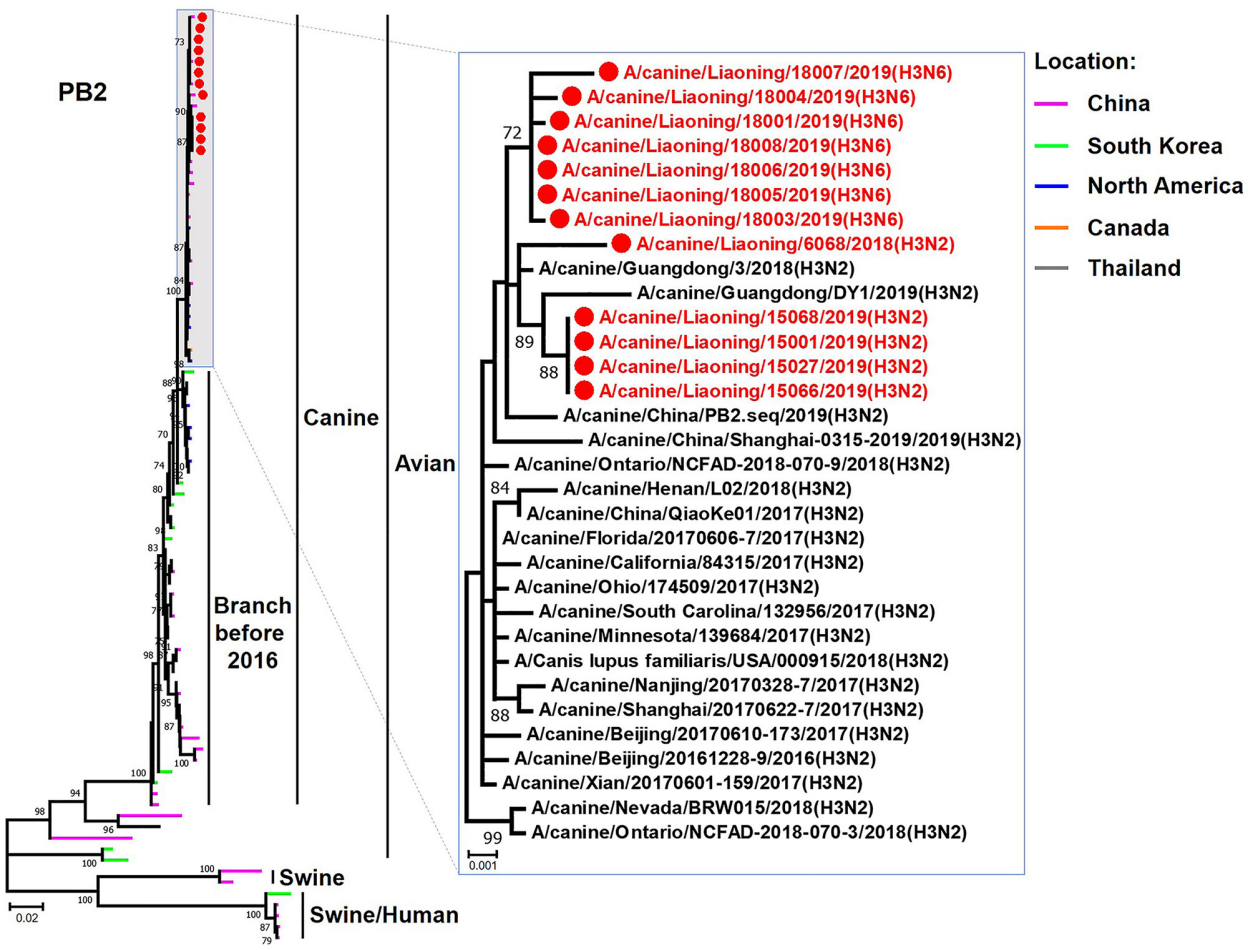
Phylogenetic analysis of the PB2 genes of H3N2 and H3N6 CIVs. It is based on nucleotide positions 28–2,307 for PB2. Isolates in this study are colored red. The scale bar indicates the number of nucleotide substitutions per site.

**Figure 4:**
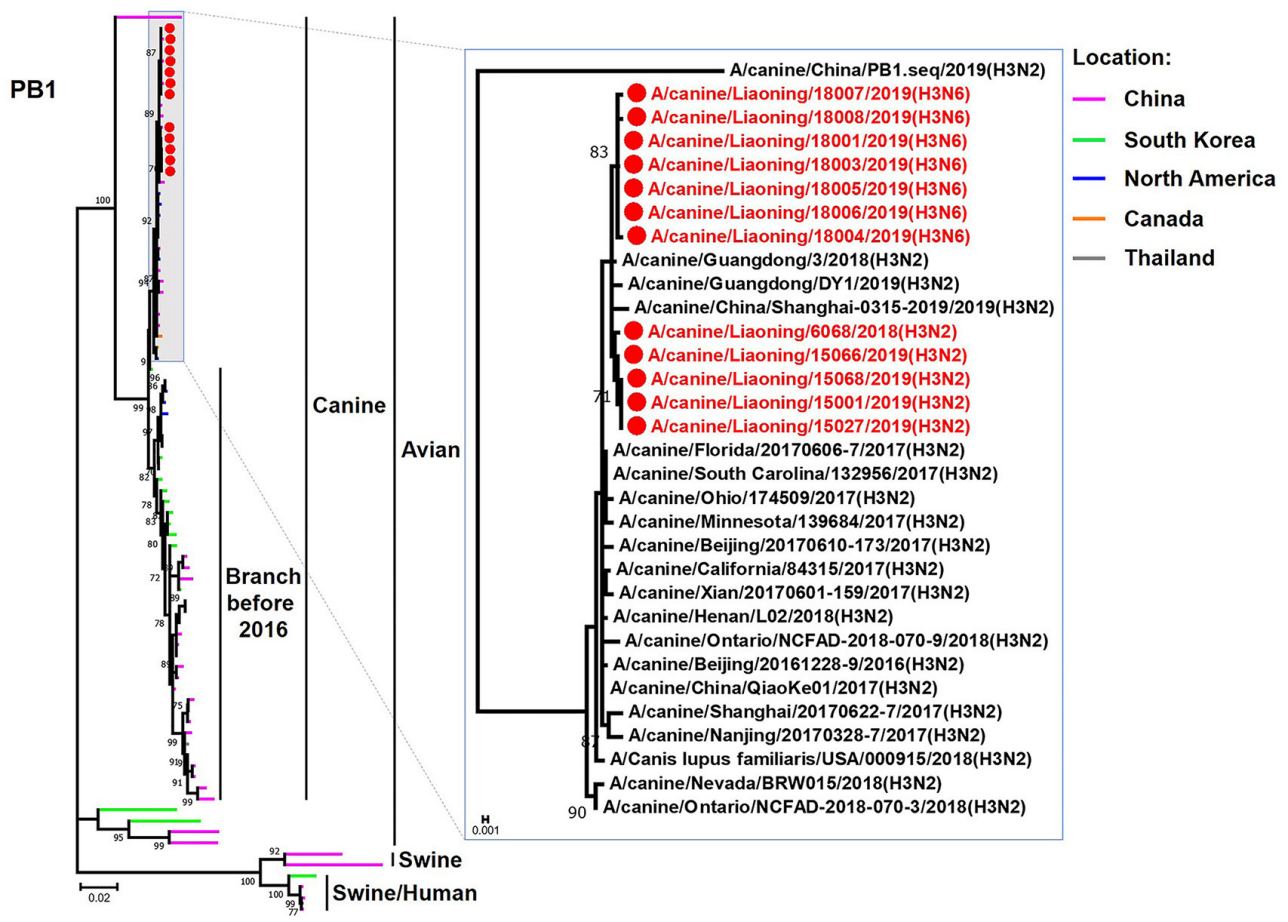
Phylogenetic analysis of the PB1 genes of H3N2 and H3N6 CIVs. It is based on nucleotide positions 25–2,298 for PB1. Isolates in this study are colored red. The scale bar indicates the number of nucleotide substitutions per site.

**Figure 5:**
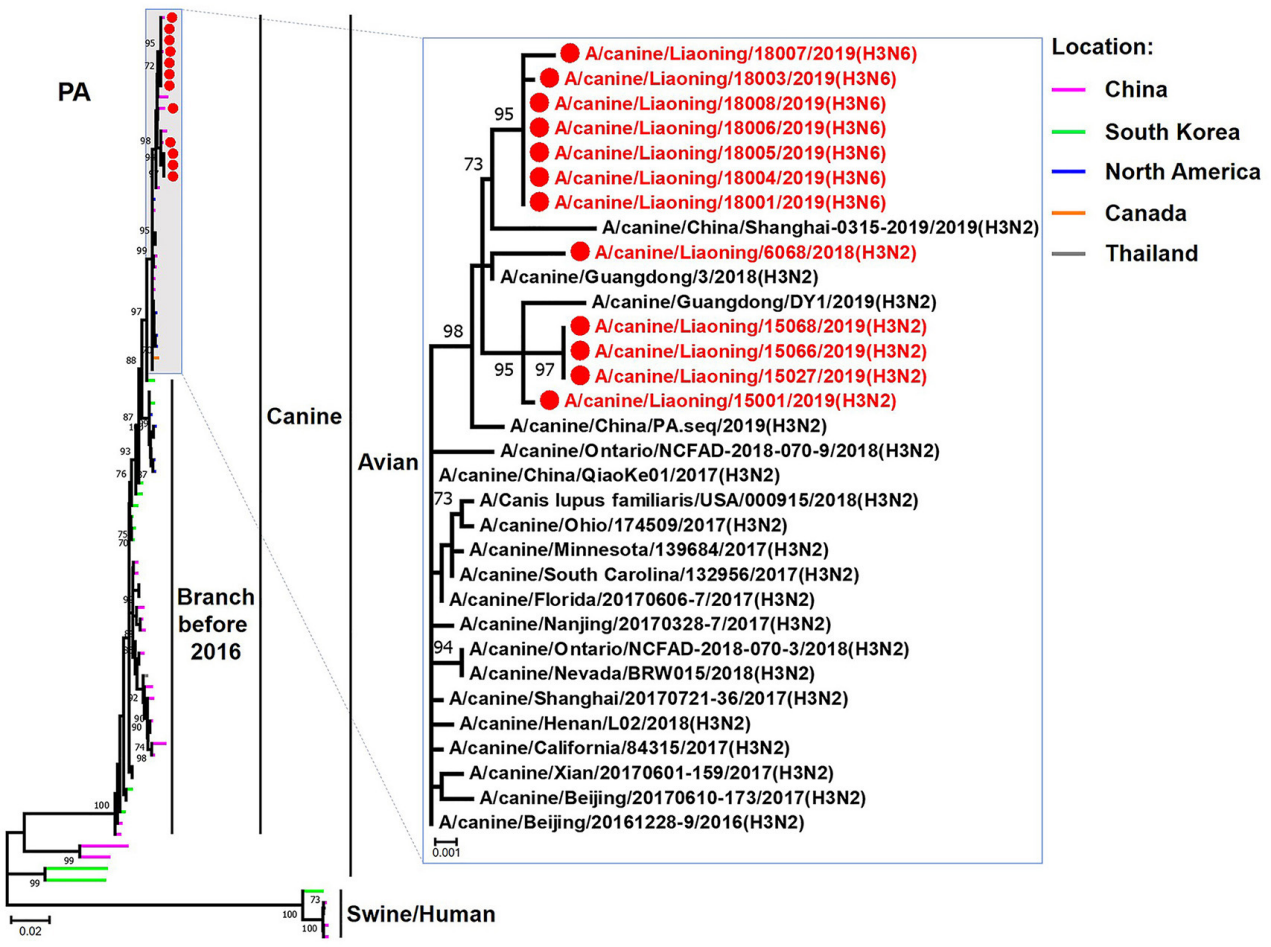
Phylogenetic analysis of the PA genes of H3N2 and H3N6 CIVs. It is based on nucleotide positions 25–2,175 for PA. Isolates in this study are colored red. The scale bar indicates the number of nucleotide substitutions per site.

**Figure 6:**
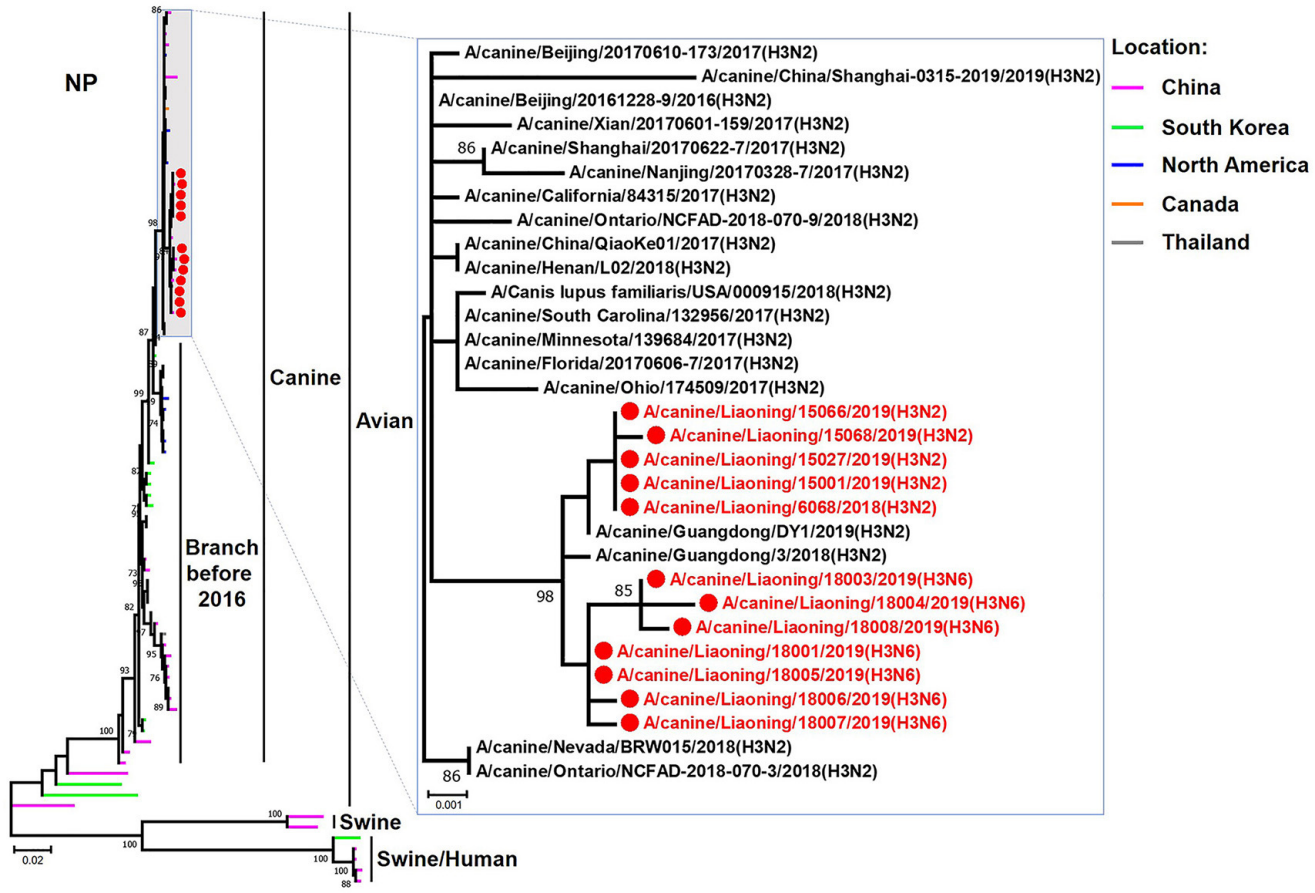
Phylogenetic analysis of the NP genes of H3N2 and H3N6 CIVs. It is based on nucleotide positions 46–1,542 for NP. Isolates in this study are colored red. The scale bar indicates the number of nucleotide substitutions per site.

**Figure 7:**
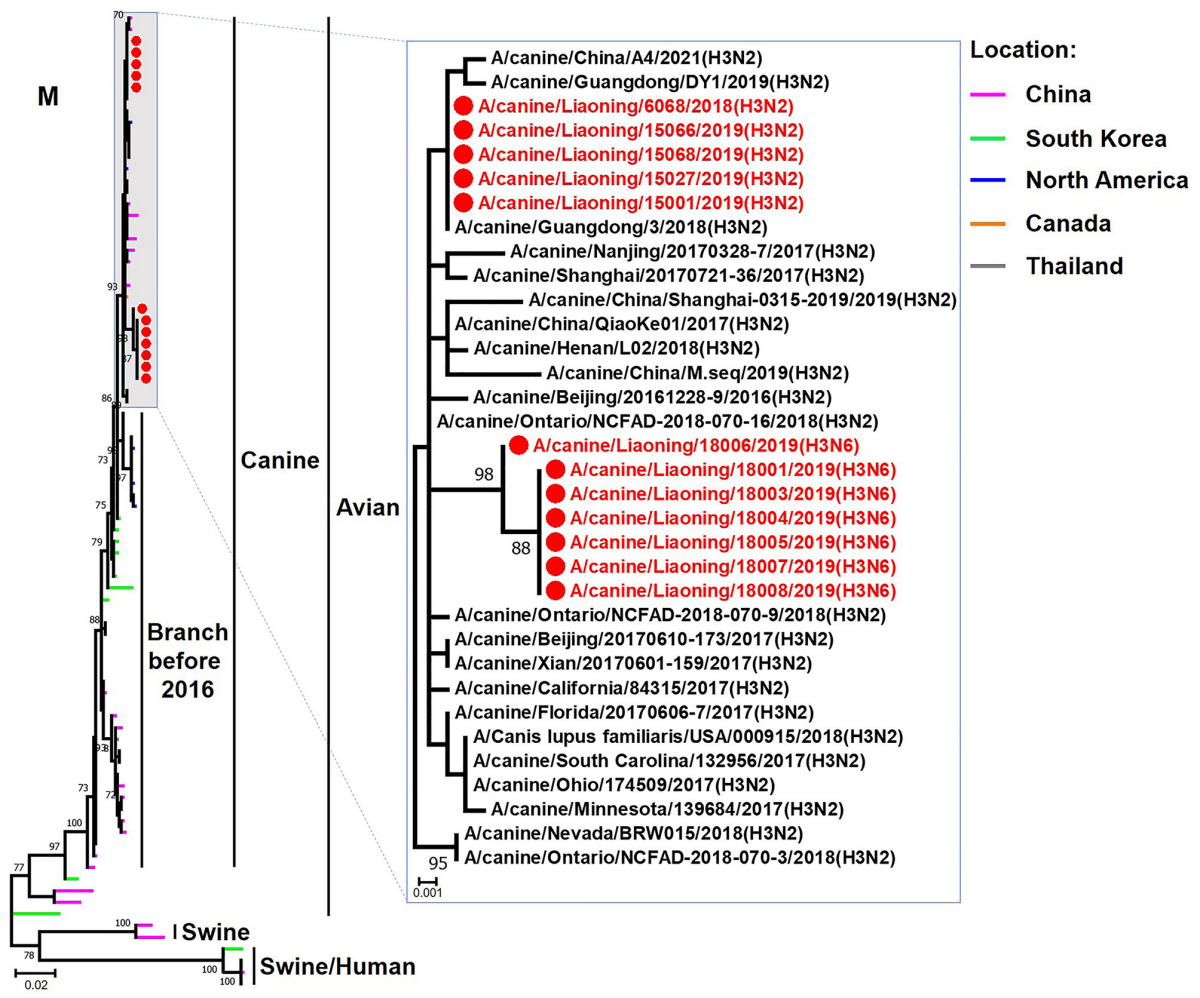
Phylogenetic analysis of the M genes of H3N2 and H3N6 CIVs. It is based on nucleotide positions 26–1,007 for M. Isolates in this study are colored red. The scale bar indicates the number of nucleotide substitutions per site.

**Figure 8:**
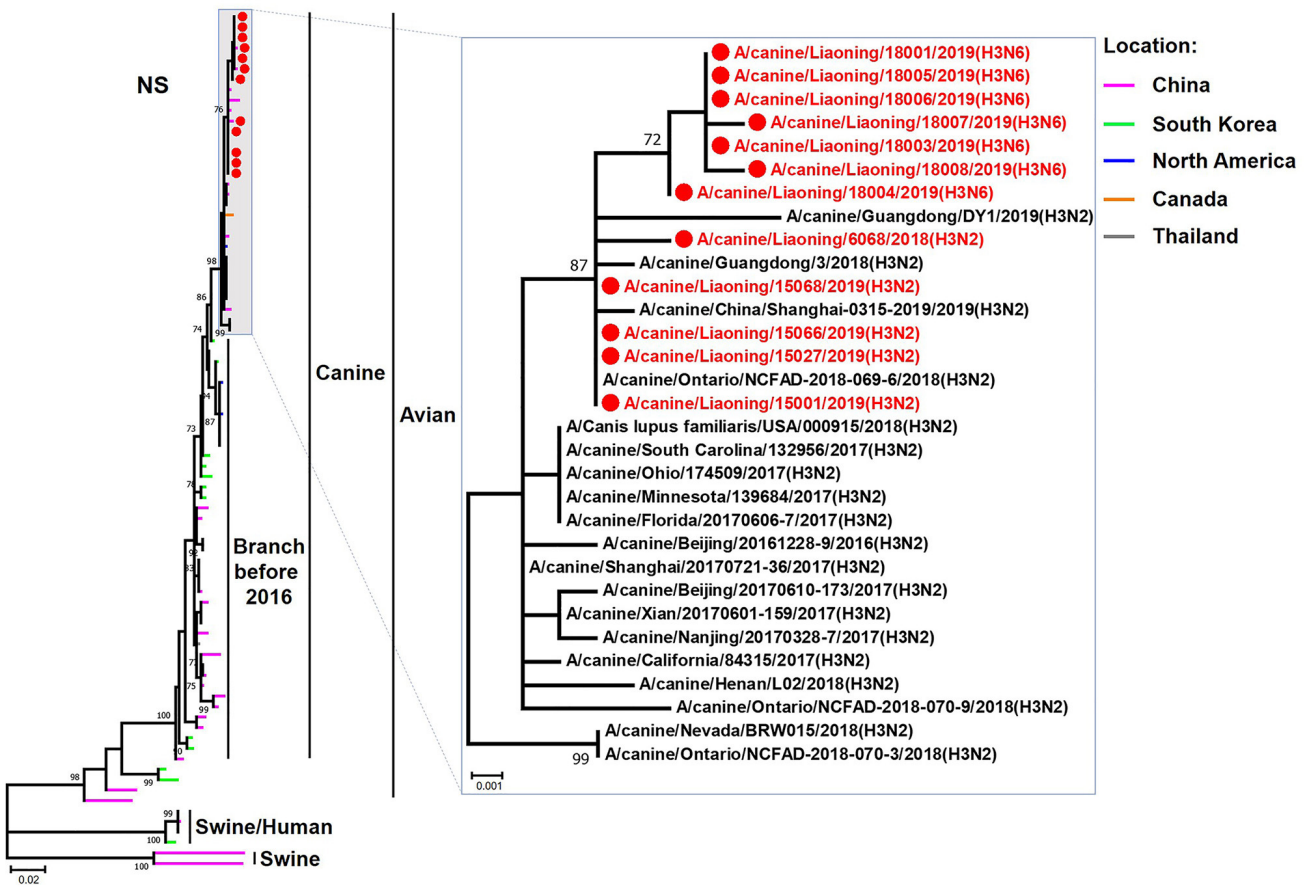
Phylogenetic analysis of the NS genes of H3N2 and H3N6 CIVs. It is based on nucleotide positions 27–864 for NS. Isolates in this study are colored red. The scale bar indicates the number of nucleotide substitutions per site.

The genome sequence of the 12 CIVs was determined by using Sanger sequencing, which revealed that of the 12 isolates, five were H3N2 (isolated on 26 November 2018 and 2 March 2019) and seven were H3N6 (isolated on 30 April 2019) CIVs (Table 2). Their nucleotide sequences have been deposited in GenBank (Accession Number: MZ323741-MZ323748, MZ323750-MZ323757, MZ323776-MZ323807, and MZ323813-MZ323860). Phylogenetic trees were constructed with MEGA X software using the maximum-likelihood (ML) method with 1000 bootstrap replications based on the sequences of the open reading frames. Except for the NA genes of the H3N6 viruses, each genome segment of the H3N2 and H3N6 viruses shared high genetic similarity (99.3%−100%) at the nucleotide level and grouped together in each phylogenetic tree. All HA genes clustered with the antigenic variants that emerged in 2016 (Figure 1). The N2 NA genes clustered with H3N2 CIVs were isolated in China, South Korea, and North America after 2016 (Figure 2). The six internal genes of all 12 isolates, the basic polymerase 2 (PB2), basic polymerase1 (PB1), acidic polymerase (PA), nucleoprotein (NP), matrix (M), and non-structural protein (NS) genes also clustered into H3N2 CIVs groups that were isolated after 2016 (Figures 3–8).

**Table 2:** Information of H3N2 and H3N6 CIVs^y^.

**Collection date (mm/dd/yy)**	**Subtypes**	**Name of isolates**	**Accession number**
11/26/18	H3N2	A/canine/Liaoning/6068/2018	MZ323741-MZ323748
03/02/19	H3N2	A/canine/Liaoning/15001/2019	MZ323750-MZ323757
03/02/19	H3N2	A/canine/Liaoning/15027/2019	MZ323776-MZ323783
03/02/19	H3N2	A/canine/Liaoning/15066/2019	MZ323784-MZ323791
03/02/19	H3N2	A/canine/Liaoning/15068/2019	MZ323792-MZ323799
04/30/19	H3N6	A/canine/Liaoning/18001/2019	MZ323800-MZ323807
04/30/19	H3N6	A/canine/Liaoning/18003/2019	MZ323813-MZ323820
04/30/19	H3N6	A/canine/Liaoning/18004/2019	MZ323821-MZ323828
04/30/19	H3N6	A/canine/Liaoning/18005/2019	MZ323837-MZ323844
04/30/19	H3N6	A/canine/Liaoning/18006/2019	MZ323829-MZ323836
04/30/19	H3N6	A/canine/Liaoning/18007/2019	MZ323845-MZ323852
04/30/19	H3N6	A/canine/Liaoning/18008/2019	MZ323853-MZ323860

^†^CIVs, canine influenza viruses.

**Figure 9:**
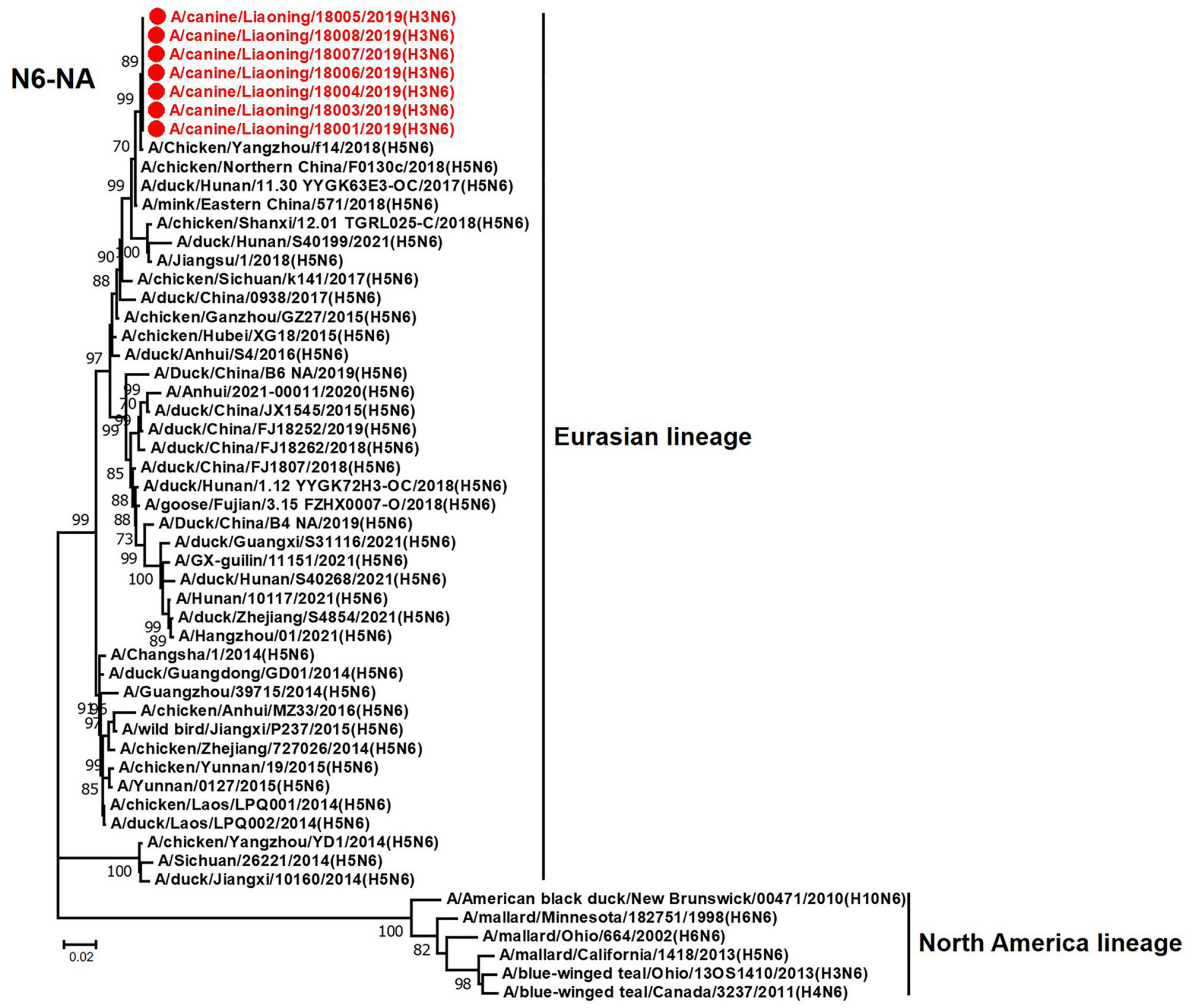
Phylogenetic analysis of the NA genes of H3N6 CIVs. It is based on nucleotide positions 19–1,398 for NA. Isolates in this study are colored red. The scale bar indicates the number of nucleotide substitutions per site.

The NA genes of the seven H3N6 CIVs shared 99.9%−100% genetic similarity at the nucleotide level and clustered with the H5N6 AIVs that were isolated in China between 2017 and 2018 (Figure 9). According to the genetic analysis results, the H3N6 CIVs represent a novel reassortant between H3N2 CIV and H5N6 AIV, probably generated from a dog co-infection case.

### 3.3. The isolates replicated differently in MDCK cells

**Figure 10:**
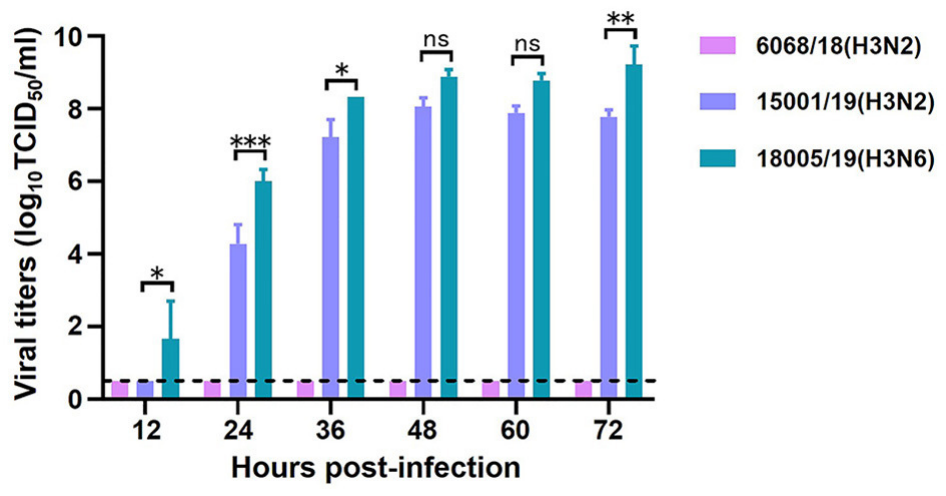
*In vitro* replication ability of the two H3N2 and one H3N6 CIVs. Growth kinetics in MDCK cells. The means of three repeats are shown, with error bars indicating the SD. Statistical analysis between different groups was performed by using a one-way analysis of variance (ANOVA) test. ^*^(*p* < 0.05), ^**^(*p* < 0.01), ^***^(*p* < 0.001), and ns (*p* > 0.05).

To evaluate the replicative ability of the CIVs, we selected two H3N2 CIVs, A/Canine/Liaoning/6068/2018 (6068/18) and A/Canine/Liaoning/15001/2019 (15001/19), and one H3N6 virus, A/Canine/Liaoning/18005/2019 (18005/19). The MDCK cells were inoculated with 10^2^ TCID_50_ of the three CIVs, and the supernatant was collected every 12 h for viral titration. Although isolated within 5 months of each other, the three CIVs showed different biologic properties. In general, the H3N6 virus replicated better than the H3N2 virus. The viral titers of 18005/19 were 10–100 times higher than those of 15001/19 at each timepoint. But 6068/18 the only virus isolated in 2018, did not replicate well in MDCK cells (Figure 10).

### 3.4. The isolates represented different replication abilities in mice

**Figure 11:**
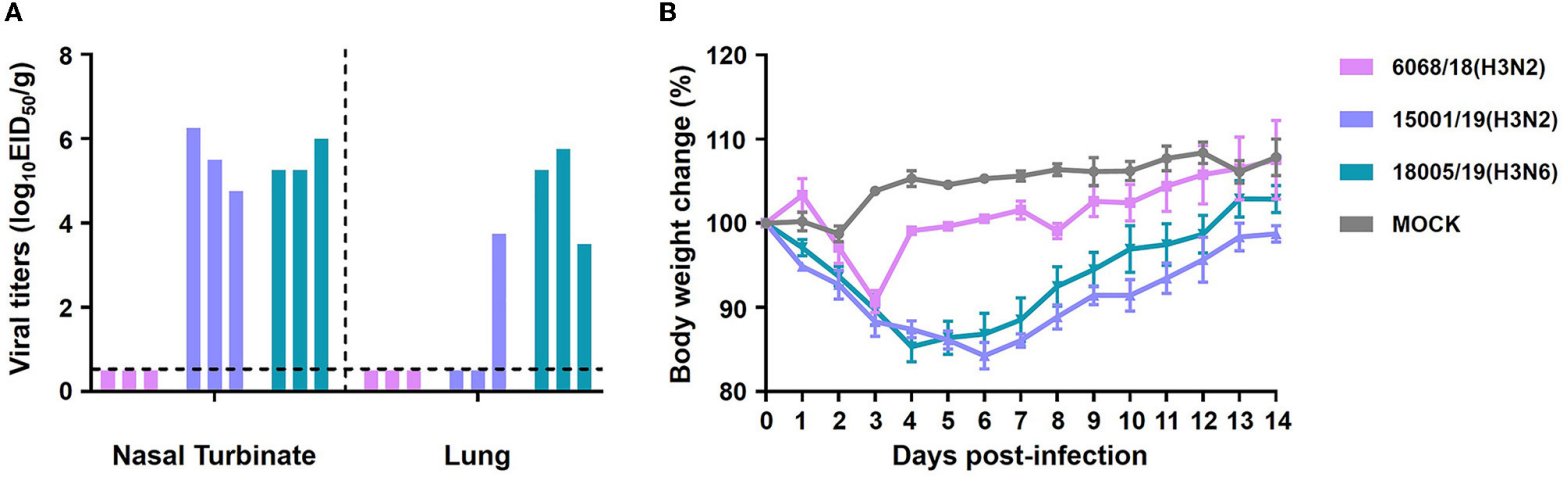
*In vivo* replication ability of the two H3N2 and one H3N6 CIVs. Replication ability in 6-week-old mice. **(A)** Mice were euthanized on day 3 p.i., and their nasal turbinates and lungs were collected and titrated for virus infectivity in eggs. **(B)** Mice were monitored for body weight loss throughout the observation period for 14 days.

We used mice as a model to evaluate the adaptation of the three CIVs in mammalian hosts. Eight 6-week-old BALB/c mice were intranasally inoculated with 10^6^ EID_50_ of each virus. Three mice from each group were euthanized on day 3 p.i., and their brains, nasal turbinates, lungs, spleens, and kidneys were collected for viral titration. The remaining mice were observed for clinical signs and body weight change until day 14 p.i. No mice exhibited significant symptoms or morbidity during the whole experiment. None of the strains were detected in the brain, spleen, or kidney of the mice (data not shown). The 15001/19 and 18005/19 viruses replicated in both the upper and lower respiratory tract of the mice (Figure 11A), although 15001/19 was only detected in one mouse lung, whereas 18005/19 was detected in the lungs of all three mice. The 6068/18 virus did not replicate well in mice (Figure 11A). Moderate, transient weight loss was observed in each inoculated group during a short period (Figure 11B).

**Figure 12:**
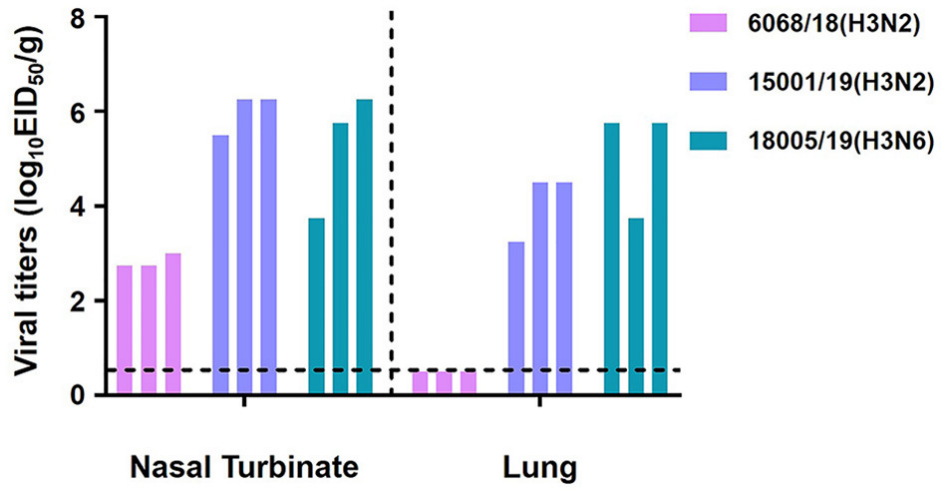
*In vivo* replication ability of the two H3N2 and one H3N6 CIVs. Replication ability in 4-week-old mice. Mice were euthanized on day 3 p.i., and their nasal turbinates and lungs were collected and titrated for virus infectivity in eggs.

We then repeated the replication study in younger animals. Three 4-week-old mice were infected with the three viruses according to the same procedure. Viral titration results indicated that the 15001/19 and 18005/19 viruses replicated more efficiently in the respiratory tracts, and 6068/18 could replicate in the upper respiratory tracts of the younger mice (Figure 12). Both 6068/18 and 15001/19 increased replication ability in younger mice. The immune system development variances of the two age groups of mice might be the reason for the 6868/18 and 15001/19 different replication phenotypes. These findings indicate that 6068/18 isolated on 26 November 2018, had not fully adapted to the mice yet, but 15001/19 had, even though 15001/19 was isolated 3 months after 6068/18.

### 3.5. The isolates exhibited avian-like receptors

**Figure 13:**
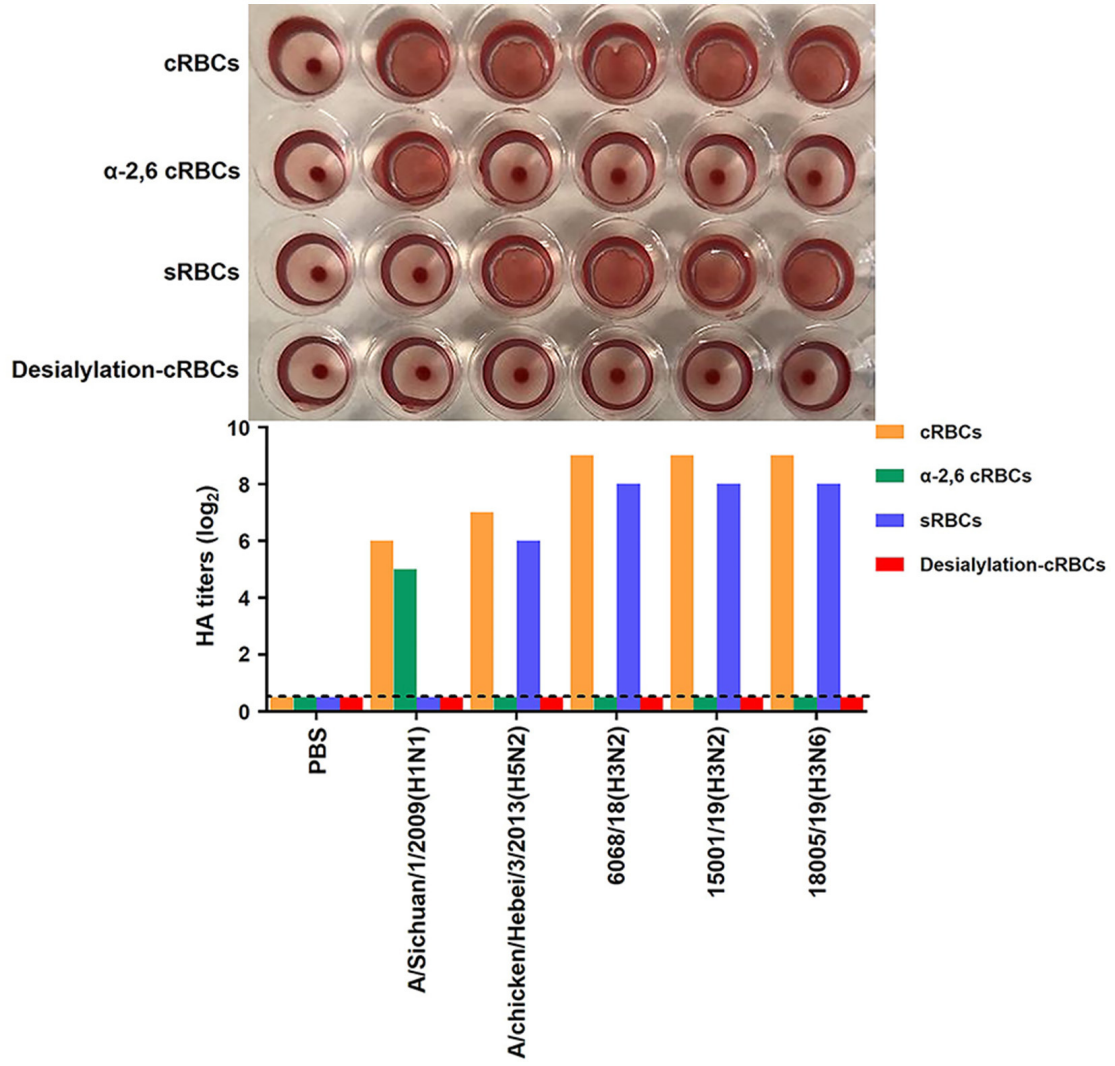
Receptor-binding analysis of the two H3N2 and one H3N6 CIVs. Agglutination activities of the H3N2 and H3N6 CIVs with various red blood cells. Four types of red blood cells: cRBCs (1% chicken red blood cells); sRBCs (1% sheep red blood cells); α-2,6-cRBCs (1% chicken red blood cells treated with α2,3-Sialidase); desialylation-cRBCs (1% chicken red blood cells treated with VCNA). The HA titer showed agglutination activities of 6068/18, 15001/19, and 18005/19 for the four types of red blood cells. PBS was a negative control group, whereas A/Sichuan/1/2009(H1N1) and A/chicken/Hebei/3/2013(H5N2) were the positive groups binding to α-2,6-cRBCs and sRBCs, respectively.

The receptor-binding property is important for influenza virus mammalian adaption. We, therefore, also evaluated the receptor-binding properties of the three CIVs, as described previously (Ito et al., 1997; Medeiros et al., 2001). The assay results demonstrated that all three CIVs bound preferentially to the avian-like receptor, α2,3-linked sialic acid (Figure 13).

## 4. Discussion

In this study, we performed active CIV surveillance in Liaoning Province for the 2018 to 2019 flu season. Twelve CIVs were isolated, including five H3N2 and seven H3N6 viruses. We systematically analyzed the genetic and biological properties of these CIVs. The phylogenetic analysis of the HA genes of 12 CIVs demonstrated that isolates in this study were all clustered into the antigenic variants emerging in 2016 (Lyu et al., 2019). NA genes of the H3N6 CIVs were clustered into H5N6 AIV groups, which were isolated in China from 2017 to 2018. There is a high possibility that the novel H3N6 CIVs are generated from H3N2 CIV and H5N6 AIV reassortment cases. H5N6 AIVs have circulated in China since 2014, and trans-species infections and reassortant cases have been reported when the H5N6 circulated in domestic poultry (Yu et al., 2015; Gu et al., 2022). Previous studies demonstrated that novel reassortant H3N6 viruses had been isolated from migratory birds and domestic birds, which contained some gene segments from the dominant H5N6 viruses (Li et al., 2020). To our knowledge, this is the first report that avian H5N6 viruses can recombine with H3N2 CIVs.

This study indicated that CIVs are evolving and adapting to mammalian hosts at an incredible speed. The H3N2 CIV isolated in November 2018 showed limited replicative ability *in vitro* and *in vivo*, but 3 months later, increased replication was detected in March 2019 H3N2 CIV isolates. Moreover, 1 month later, novel reassortant H3N6 CIVs appeared in April 2019. The H3N6 reassortant possessed enhanced mammalian adaption ability compared with H3N2 CIVs.

Genetic mutation and reassortment were both observed among our CIVs isolates. There were only five amino acid differences between 6068/18 and 15001/19, PB2-A292T, PB2-K578E, PA-I561M, HA-G271D (H3 numbering), and NS1-G142E. The effects of these substitutions on the biological properties of these viruses require further exploration. The alignment of the N6 NA stalk showed that there was an 11 amino acid deletion at positions 58 to 68, which had been identified in H5N6 AIVs. According to the *in vitro* and *in vivo* replication study results, it seemed that the H3N2 HA and H5N6 NA genes of the novel CIV are more functionally and optimally cooperative than the HA and NA genes of H3N2 CIVs. Genome reassortment between different subtypes of flu strains is an important driving force for the evolution of influenza viruses and the sources of potential pandemic strains. Three out of four human influenza pandemics of the last century were caused by reassortant viruses (Li and Chen, 2014).

As companion animals, dogs are closely related to humans, and their potential threat to public health deserves high attention. In this study, we found that H3 CIVs were undergoing an evolution process at an incredible speed and provided important information about the evolutionary status of H3 CIVs. Further surveillance and risk analysis of CIVs should be performed.

## Data availability statement

The original contributions presented in the study are included in the article/Supplementary material, further inquiries can be directed to the corresponding authors.

## Ethics statement

The animal study was reviewed and approved by the mice experiments were approved for such use by the Animal Experimentation and Laboratory Animal Welfare Committee of Shenyang Agricultural University (No. 202106009, approved on 8 March 2021).

## Author contributions

BM performed the experiments, analyzed the data, and wrote the first draft of the manuscript. HL, YF, and DZ collected the canine samples. CF and WG assisted with virus isolation and animal experiments. HC and YZ guided the experiments, conceived the study, analyzed the data, and finalized the manuscript. All authors contributed to the article and approved the submitted version.
